# Inverse association between central obesity and arterial stiffness in Korean subjects with metabolic syndrome: a cross-sectional cohort study

**DOI:** 10.1186/1758-5996-7-3

**Published:** 2015-01-27

**Authors:** Ki-Bum Won, Hyuk-Jae Chang, Hiroyuki Niinuma, Koichiro Niwa, Kyewon Jeon, In-Jeong Cho, Chi-Young Shim, Geu-Ru Hong, Namsik Chung

**Affiliations:** Division of Cardiology, Yonsei Cardiovascular Center, Yonsei University College of Medicine, 50 Yonsei-ro, Seodaemungu, Seoul, 120-752 Republic of Korea; Division of Cardiology, St. Luke’s International Hospital, Tokyo, Japan; Severance Biomedical Science Institute, Seoul, Republic of Korea

**Keywords:** Metabolic syndrome, Central obesity, Pulse wave velocity

## Abstract

**Background:**

Metabolic syndrome (MetS) is associated with increased risks of diabetes and atherosclerotic cardiovascular disease. Whether central obesity (CeO) is a prerequisite for the diagnosis of MetS in the International Diabetes Federation (IDF) definition is a substantial issue because it may influence the clinical value of MetS for predicting subclinical atherosclerosis.

**Methods:**

We investigated the relation between MetS, as defined by the National Cholesterol Education Program–Adult Treatment Panel (NCEP–ATP) III criteria, and arterial stiffness according to CeO status in 2,560 healthy Korean subjects who participated in a community-based cohort study. Arterial stiffness was measured using brachial-ankle pulse wave velocity (baPWV).

**Results:**

The prevalence of MetS was 37%; 84% of MetS subjects had CeO. The prevalence of diabetes was significantly higher in MetS subjects than in non-MetS subjects (30 vs. 8%, p <0.001). The number of MetS components was significantly correlated with baPWV (r = 0.311, p <0.001). In a subgroup analysis of MetS subjects, the prevalence of diabetes was not significantly different in MetS subjects with and without CeO. MetS subjects without CeO had significantly higher baPWV than those with CeO (1654 ± 315 vs. 1578 ± 270 cm/s, p = 0.002). Multiple regression models revealed that waist circumference was independently associated with decreased baPWV in MetS subjects.

**Conclusions:**

Despite the significant correlation between the number of MetS components and arterial stiffness, there appeared to be an inverse association between CeO and arterial stiffness in MetS subjects. In contrast to the IDF definition, our findings suggest that CeO is not crucial for the diagnosis of MetS in otherwise healthy Koreans having multiple metabolic risk factors with respect to subclinical atherosclerosis reflected in arterial stiffness.

## Background

Metabolic syndrome (MetS) is associated with increased risks of diabetes and atherosclerotic cardiovascular disease (CVD) [1–3]. The prevalence of MetS is rapidly increasing worldwide [4, 5], and the diagnosis of MetS is important to identify individuals at high risk for CVD because MetS is strongly associated with the development of atherosclerotic major cardiovascular (CV) events [6, 7]. Although the pathogenesis of MetS related to its individual components is complex, central obesity (CeO) has been considered to be a causative factor of MetS because it is a predominant component of MetS [8, 9]. Especially, the International Diabetes Federation (IDF) suggests that CeO, defined by waist circumference with ethnicity-specific values, is essential for the diagnosis of MetS [10]. However, subjects without CeO but with multiple metabolic abnormalities are not diagnosed with MetS according to the IDF definition.

MetS is a concept that focuses attention on a constellation of complex, multifactorial health problems for the prevention of CVD. Although obesity is one of the important risk factors for the development of CVD [11, 12], several recent studies have reported the phenomenon of the “obesity paradox” in which obesity is associated with improved prognoses in patients after the development of CVD, including acute myocardial infarction (AMI) and heart failure [13–16]. These results may imply that, in a healthy population, the primary prevention of CVD might be more important in non-obese individuals with multiple CV risk factors. Thus, it is important to identify the significance of MetS diagnosis in subjects without CeO with respect to subclinical atherosclerosis in a relatively healthy population without a previous history of major CV events.

Arterial stiffness is an important surrogate marker of subclinical atherosclerosis, and increasing arterial stiffness represents an increased risk for major adverse CV events [17, 18]. Brachial-ankle pulse wave velocity (baPWV) is a reproducible index of arterial elasticity and stiffness [19]. Although a previous study reported that MetS is associated with increased arterial stiffness [20], the association between MetS and arterial stiffness according to CeO status is unknown. Thus, the present study investigated the relation between MetS and arterial stiffness according to the presence of CeO in relatively healthy Korean subjects who participated in health examinations for a community-based cohort study.

## Methods

This is a cross-sectional investigation analyzing data collected for a prospective cohort study. We used the data of 2,560 subjects who participated in baseline health examinations for a community-based cohort study in the Seoul area between April 2010 and November 2012. Subjects with a previous history of CVD, cerebrovascular disease, or malignancy were not included in the present study according to the study protocol.

All blood samples were obtained after an 8-hr fast and analyzed for glucose, triglycerides, high-density lipoprotein (HDL) cholesterol, and low-density lipoprotein (LDL) cholesterol. Weight, height, and waist circumference were measured while subjects wore light clothing and no shoes. Waist circumference was measured at the midpoint between the iliac crest and the lower border of the rib cage. Body mass index (BMI) was calculated as weight (kg) ÷ height (m^2^). Diabetes was defined as either fasting glucose ≥126 mg/dL, a referral diagnosis of diabetes, or antidiabetic treatment. MetS was defined as the presence of 3 or more of the following components, based on the National Cholesterol Education Program–Adult Treatment Panel (NCEP–ATP) III definition [1]: (a) CeO based on waist circumference ≥ 90 cm in males or ≥ 80 cm in females in accordance with the Asia Pacific World Health Organization guidelines; (b) HDL cholesterol <40 mg/dL in males or <50 mg/dL in females; (c) triglycerides ≥150 mg/dL; (d) systolic blood pressure (SBP) ≥130 mmHg or diastolic blood pressure (DBP) ≥85 mmHg or on antihypertensive treatment; and (e) impaired fasting glucose, defined as fasting glucose ≥100 mg/dL, or antidiabetic treatment.

All subjects abstained from caffeine-containing food for at least 45 minutes before the measurement of baPWV. After all subjects had been resting in the supine position for at least 5 minutes in a quiet room, blood pressure and baPWV were measured using an automated waveform analyzer (Colin VP-2000, Colin Medical Instruments Corp., Komaki, Japan). Pneumatic cuffs were wrapped around both upper arms and ankles and connected to a plethysmographic sensor to determine the volume pulse waveform. The highest value of baPWV measured on either side of each patient was used for analysis. This study was approved by the local ethics committee of our institution, and informed consent was obtained from each participant.

### Statistical analysis

Continuous variables are expressed as mean ± SD or medians and interquartile range according to the distribution. Categorical variables are presented as percentages. Continuous variables were compared using independent t-test or Mann–Whitney U-test, and categorical variables were compared using the χ^2^ test or Fisher’s exact test, as appropriate. Correlational analysis between the number of MetS components and baPWV was performed using Pearson’s correlation test. After identifying the differences in baPWV according to the presence of MetS, the mean value of baPWV was compared in subjects with MetS according to their CeO status. Univariate linear regression analysis was performed to evaluate the association between CeO and baPWV in subjects with MetS. Multiple regression models were analyzed to identify the independent association between CeO and baPWV after consecutive adjustment for confounding risk factors, including age, gender, smoking, heart rate, serum creatinine, number of MetS components, and other MetS components in subjects with MetS. Multiple linear regression analysis for identifying the association between baPWV and CV risk factors was performed in subjects with MetS. The forced entry method was used to enter independent variables into the multiple regression models. SPSS version 18 (SPSS Inc., Chicago, IL, USA) was used for all statistical analyses. All statistical tests were 2-tailed, and p <0.05 was considered significant.

## Results

**Figure 1:**
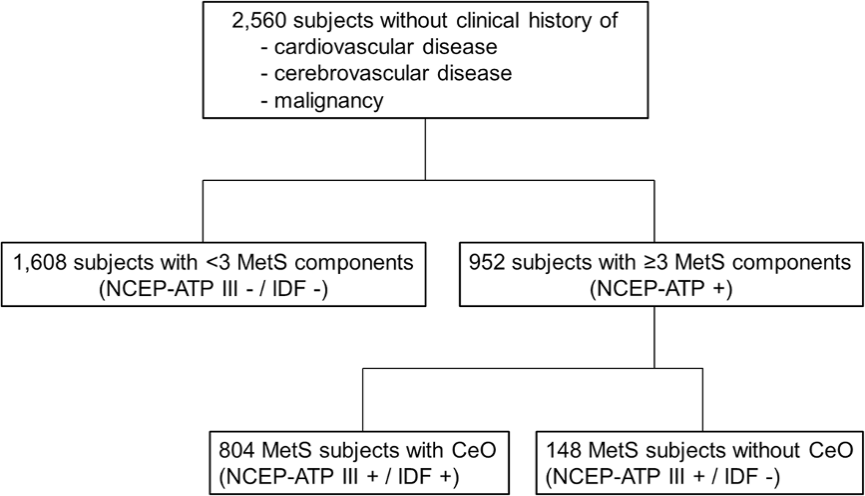
**Study flowchart of the present cohort study.**

**Figure 2:**
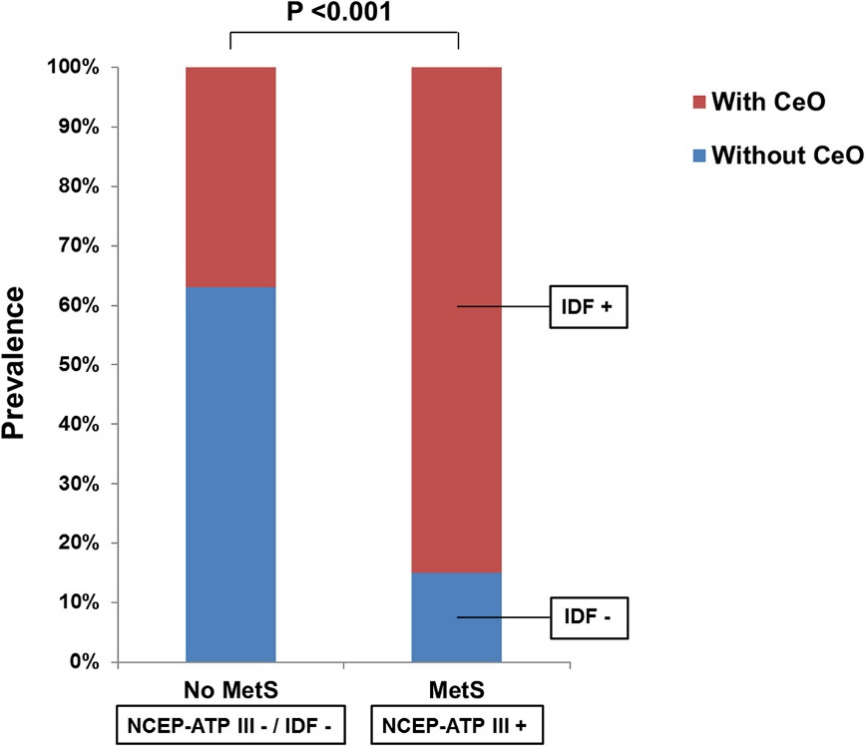
**Incidence of CeO in subjects with and without MetS.**

**Figure 3:**
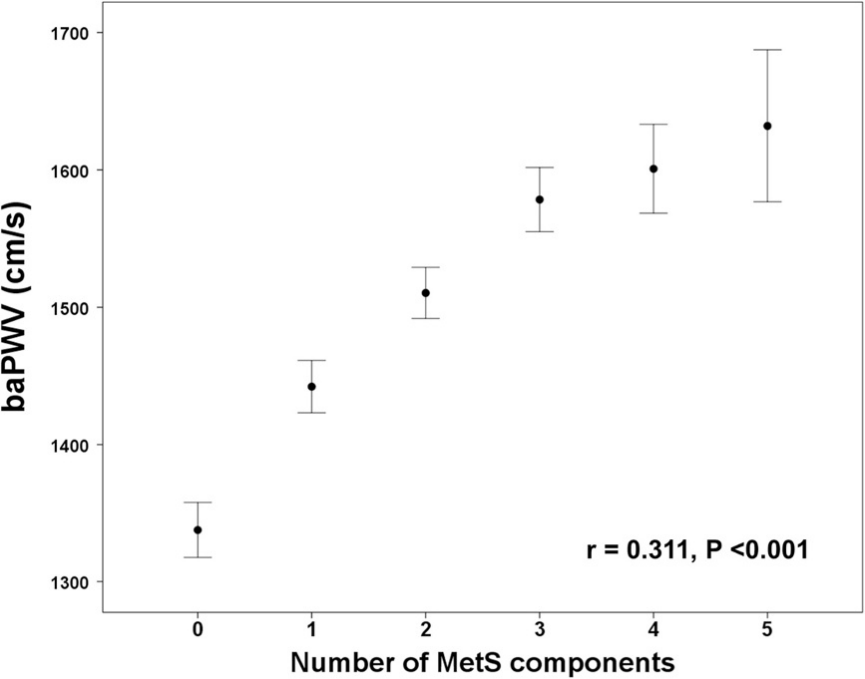
**Correlation between the number of MetS components and baPWV.** The error bars represent the standard deviation.

A flowchart of this study is presented in Figure 1. The clinical characteristics of the 2,560 participants (60 ± 8 years, 33% men) in this study are shown in Table 1. The prevalence of MetS was 37%; 84% of subjects with MetS had CeO that satisfied the IDF definition. The incidence of CeO was significantly higher in subjects with MetS than in those without MetS (84 vs. 37%, p <0.001) (Figure 2). The prevalence of diabetes was significantly higher in subjects with MetS than in those without MetS (30 vs. 8%, p <0.001). The number of MetS components was significantly correlated with baPWV (r = 0.311, p <0.001) (Figure 3).

**Figure 4:**
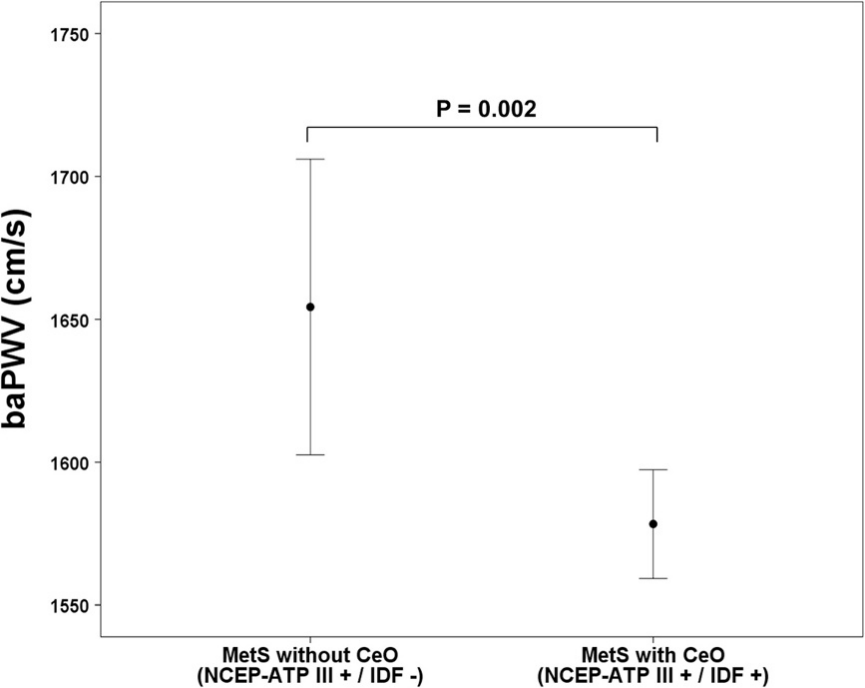
**Comparison of baPWV in subjects with MetS according to CeO status.** The error bars represent the standard deviation.

Among the subjects with MetS, the prevalence of diabetes was not significantly different between MetS subjects with and without CeO. The incidence of current medications for the treatment of hypertension, dyslipidemia, and diabetes was not significantly different between MetS subjects with and without CeO. However, the incidence of male gender (62 vs. 32%, p <0.001) and smoking (52 vs. 29%, p <0.001) was significantly higher in MetS subjects without CeO than in those with CeO. MetS subjects without CeO had significantly higher baPWV than those with CeO (1654 ± 315 vs. 1578 ± 270 cm/s, p = 0.002) (Figure 4).

**Table 1:** **Baseline characteristics**

Characteristics	No MetS (NCEP-ATP III - / IDF -) (n = 1,608)	MetS (NCEP-ATP III +)			P
		Total (n = 952)	With CeO (IDF +) (n = 804)	Without CeO (IDF -) (n = 148)	
Age (years)	65 (60, 69)	64 (57, 69)	62 ± 8	61 ± 7	<0.001
Male gender (%)	31	36	32	62*	0.004
Smoking (%)	24	32	29	52*	<0.001
BMI (kg/m^2^)	24.0 ± 2.7	26.4 ± 2.9	26.8 ± 2.8	23.9 ± 1.9*	<0.001
Waist circumference (cm)	81 ± 8	89 ± 7	90 ± 7	81 ± 6*	<0.001
Heart rate (bpm)	66 ± 9	68 ± 10	68 ± 10	70 ± 11^†^	<0.001
SBP (mmHg)	120 ± 14	128 ± 15	128 ± 15	130 ± 15	<0.001
DBP (mmHg)	72 ± 9	77 ± 10	76 ± 10	79 ± 10^†^	<0.001
Antihypertensive medications (%)	29	66	67	66	<0.001
Total cholesterol (mg/dL)	201 ± 36	197 ± 37	197 ± 37	195 ± 36	0.006
Triglycerides (mg/dL)	94 (71, 118)	136 (103, 186)	164 ± 82	210 ± 80*	<0.001
HDL cholesterol (mg/dL)	57 (51, 63)	45 (37, 55)	47 ± 13	41 ± 11*	<0.001
LDL cholesterol (mg/dL)	110 (83, 127)	106 (83, 130)	121 ± 34	117 ± 33	0.096
Lipid lowering medications (%)	27	31	31	27	0.034
Creatinine (mg/dL)	0.78 (0.63, 0.95)	0.81 (0.68, 0.93)	0.79 ± 0.19	0.87 ± 0.19*	<0.001
Fasting glucose (mg/dL)	121 (105, 137)	125 (108, 143)	109 ± 24	114 ± 27^‡^	<0.001
Diabetes (%)	8	30	30	30	<0.001
Antidiabetic treatment (%)	7	25	82	84	<0.001
Duration of diabetes (months)	92 (44, 202)	92 (44, 152)	110 ± 85	107 ± 87	0.177
baPWV (cm/s)	1576 (1414, 1760)	1625 (1452, 1864)	1578 ± 270	1654 ± 315^†^	<0.001

Continuous variables are expressed as mean ± *SD* or median **[**interquartile]. Categorical variables are presented as percentages. baPWV, brachial-ankle pulse wave velocity; *BMI*, body mass index; *CeO*, central obesity; *DBP*, diastolic blood pressure; *HDL*, high-density lipoprotein; *IDF*, International Diabetes Federation; LDL, low-density lipoprotein; MetS, metabolic syndrome; *NCEP-ATP III*, National Cholesterol Education Program-Adult Treatment Panel III; *SBP*, systolic blood pressure. *p <0.001 vs. NCEP-ATP III + / IDF +, ^†^p <0.01 vs. NCEP-ATP III + / IDF +, ^‡^p <0.05 vs. NCEP-ATP III + / IDF +.

Multiple linear regression models were performed to identify the association between CeO and baPWV in subjects with MetS. Unadjusted linear regression analysis showed an inverse association between central obesity and baPWV in subjects with MetS (B = -76.001, p <0.001). This inverse association persisted after consecutive adjustment for confounding risk factors: age (B = -88.328, p <0.001); age and gender (B = -85.560, p <0.001); age, gender, and smoking (B = -85.537, p <0.001); age, gender, smoking, and heart rate (B = -70.866, p = 0.001); age, gender, smoking, heart rate, and serum creatinine (B = -70.392, p = 0.002); age, gender, smoking, heart rate, serum creatinine, and number of MetS components (B = -78.961, p = 0.001); and age, gender, smoking, heart rate, LDL, eGFR, number of MetS components, and other MetS components (B = -84.231, p = 0.001) (Table 2).

**Table 2:** **Multiple regression models for identifying the association between central obesity and baPWV in subjects with MetS**

	baPWV				
	R ^2^	B	SE	β	P
**MetS with CeO**					
Model 1	0.010	-76.001	24.842	-0.099	<0.001
Model 2	0.191	-88.328	22.480	-0.115	<0.001
Model 3	0.191	-85.560	23.077	-0.111	<0.001
Model 4	0.191	-85.537	23.090	-0.111	<0.001
Model 5	0.257	-70.866	22.208	-0.092	0.001
Model 6	0.258	-70.392	22.207	-0.091	0.002
Model 7	0.260	-78.961	22.741	-0.103	0.001
Model 8	0.285	-84.231	25.272	-0.109	0.001

β**,** standardized coefficients; B, unstandardized coefficients; baPWV, brachial-ankle pulse wave velocity; CeO, central obesity; LDL, low-density lipoprotein; MetS, metabolic syndrome; SE, standard error.

“Other MetS components” refer to the dichotomous variables of HDL cholesterol, triglycerides, blood pressure, and fasting glucose according to the NCEP–ATP III criteria.

Model 1: Unadjusted.

Model 2: Adjusted for age.

Model 3: Adjusted for age and male gender.

Model 4: Adjusted for age, male gender, and smoking.

Model 5: Adjusted for age, male gender, smoking, and heart rate.

Model 6: Adjusted for age, male gender, smoking, heart rate, and serum creatinine.

Model 7: Adjusted for age, male gender, smoking, heart rate, serum creatinine, and number of MetS components.

Model 8: Adjusted for age, male gender, smoking, heart rate, serum creatinine, number of MetS components, and other MetS components.

Multiple linear regression analysis to identify the association between baPWV and CV risk factors was performed in subjects with MetS. Waist circumference (B = -4.536, p <0.001), age (B = 15.973, p <0.001), heart rate (B = 6.425, p <0.001), fasting glucose (B = 0.648, p = 0.021), and SBP (B = 9.166, p <0.001) were independently associated with baPWV in subjects with MetS (Table 3).

**Table 3:** **Multiple linear regression analysis for identifying the association between baPWV and CV risk factors in subjects with MetS**

	baPWV			
	B	SE	β	P
R^2^ = 0.508				
Waist circumference	-4.536	1.025	-0.117	<0.001
Age	15.973	0.911	0.429	<0.001
Male gender	-15.440	25.512	-0.027	0.545
Smoking	18.800	22.330	0.032	0.400
Heart rate	6.425	0.683	0.229	<0.001
Serum creatinine	66.219	44.050	0.046	0.133
Number of MetS components	2.052	11.565	0.005	0.859
Triglycerides	0.115	0.089	0.034	0.198
HDL cholesterol	0.707	0.599	0.032	0.238
Fasting glucose	0.648	0.281	0.057	0.021
SBP	9.166	0.644	0.494	<0.001
DBP	0.169	1.031	0.006	0.870

β**,** standardized coefficients; B, unstandardized coefficients; baPWV, brachial-ankle pulse wave velocity; *DBP*, diastolic blood pressure; *HDL*, high-density lipoprotein; MetS, metabolic syndrome; *SBP*, systolic blood pressure; *SE*, standard error.

## Discussion

To the best of our knowledge, the present study provides the first information on the inverse association between CeO and arterial stiffness in MetS subjects among a relatively healthy Korean population without major adverse CV events. In addition, we identified the prevalence of diabetes was not significantly different in MetS subjects according to their CeO status. It might be reasonable to diagnose MetS based on the number of metabolic abnormalities considering the significant correlation between the number of MetS components and the progression of subclinical atherosclerosis that was reflected in arterial stiffness. However, in contrast with the IDF definition, our results indicate that the diagnosis of MetS should be necessary in subjects without CeO but with multiple metabolic risk factors with respect to subclinical atherosclerosis reflected in arterial stiffness in the general population.

MetS is a concept that focuses attention on complex multifactorial health problems for the prevention of CVD. The prevalence of MetS is rapidly increasing worldwide, and it affects approximately 31% of adults in Korea [21]. This simultaneous clustering of metabolic abnormalities appears to confer a substantial additional CV risk over and above the sum of the risk associated with each individual abnormality [22, 23]. Thus, MetS has been promoted as a means for identifying the risk of CVD development in clinical practice. Although the pathogenesis of MetS related to the individual components is not well understood, CeO has been considered to have a pivotal role in the pathogenesis of MetS [8, 9]. In particular, compared with other definitions of MetS, the IDF criteria defined CeO as a prerequisite for the diagnosis of MetS [10]. However, individuals without CeO but having multiple metabolic abnormalities are not diagnosed with MetS according to the IDF definition. Because this may influence the clinical usefulness of MetS for the prevention of CVD, it is an important issue whether CeO is a prerequisite for the diagnosis of MetS.

Arterial stiffness is a significant surrogate marker of subclinical atherosclerosis, and increasing arterial stiffness is independently associated with an increased risk for major adverse CV events [17, 18]. Although previous studies reported that MetS is strongly associated with subclinical atherosclerosis [20, 24], the relation between MetS and subclinical atherosclerosis according to the presence of CeO has not been evaluated. In this study, we evaluated the correlation between the number of MetS components and arterial stiffness. Then, we compared the arterial stiffness in subjects with MetS defined by NCEP–ATP III criteria according to their CeO status. Interestingly, despite the significant correlation between the number of MetS components and arterial stiffness, MetS subjects without CeO had significantly increased arterial stiffness compared to those with CeO. This inverse association persisted after adjusting for confounding CV risk factors including other MetS components. Considering these results, it might be reasonable to diagnose MetS based solely on the number of metabolic risk factors, but the diagnosis of MetS might be even more important in subjects without CeO but with multiple metabolic risk factors in order to identify individuals at high risk for CVD. The IDF definition may be helpful for understanding the pathogenesis of MetS because this definition primarily focuses on the predominant characteristic of MetS, but it is uncertain whether the IDF definition is more efficient for predicting subclinical atherosclerosis in the general population compared to other definitions of MetS. The results of our study might raise an important question as to whether CeO should be a prerequisite component for the diagnosis of MetS for the prevention of CVD in the general population.

Clinical features of atypical MetS subjects who are not accompanied by CeO have not been well known, especially in Asian populations. Most definitions of MetS are reasonable, in that the criteria for metabolic abnormalities used to identify individuals with MetS differ according to gender and ethnicity. However, other lifestyle factors strongly related to the development of CVD such as smoking status are not considered in the definition of MetS. The relation between smoking and obesity is not completely understood, but previous numerous cross-sectional studies have reported that body weight or BMI is lower in smokers than in nonsmokers [25–29]. Moreover, smokers weighed less than nonsmokers, and body leanness increased with the duration of smoking in the second National Health and Nutrition Examination Survey study [30]. Although these studies did not evaluate the association between smoking and CeO, smoking might have a suppressive effect on CeO considering the close association among anthropometric indices including body weight, BMI, and waist circumference. In the present study, while the incidence of smoking was significantly higher in subjects with MetS than in those without MetS, MetS subjects without CeO had significantly higher incidence of smoking than those with CeO. Considering these results, further investigation to identify the impact of smoking on MetS and its individual components may be necessary.

Although obesity is significantly associated with the development of CVD [11, 12], an interesting phenomenon known as the “obesity paradox” has been reported in patients after the development of CVD. Several studies reported either an inverse linear or U-shaped association between BMI and all-cause mortality in patients with heart failure [31–33]. This association was also replicated in patients after the event of AMI [15, 16, 34]. The exact mechanism by which obesity may improve prognoses in patients with heart failure or AMI is unknown. Moreover, the impact of CeO on prognoses in patients with major CV complications has not been evaluated despite the significant relationships among anthropometric indices. However, considering this paradoxical phenomenon, is note worthy that the primary prevention of major CVD may need to be emphasized more in high-risk, otherwise healthy, non-obese subjects who have no previous history of major adverse CV events. Recently, the World Health Organization (WHO) strongly recommended that the concept of MetS should be applied in subjects without established CVD [35]. This community-based cohort study was performed in subjects who had no previous history of CVD, cerebrovascular disease, or malignancy. We identified the clinical features of atypical MetS subjects who did not have CeO compared to the typical MetS subjects with CeO, and found that the IDF definition had a major limitation for predicting subclinical atherosclerosis compared to the NCEP–ATP III definition in the present study.

MetS has been promoted as a means of identifying the risk for type 2 diabetes development. While the pathogenesis of MetS is not well understood, CeO and insulin resistance are acknowledged as important causative factors for the development of MetS [8–10]. It is obvious that CeO is strongly associated with insulin resistance. However, despite the substantial increase in the prevalence of MetS and type 2 diabetes in Asian population, the clinical features of type 2 diabetes in Asia are distinctly different from the features of type 2 diabetes in other parts of the world; it develops in a much shorter time, at a younger age, and in subjects with much lower BMI in Asia [36]. In addition, several studies on the pathogenesis of type 2 diabetes in Korean subjects reported that impaired insulin secretion was more prominent than insulin resistance, even in people with impaired glucose tolerance [37, 38]. In the present study, the incidence of CeO was significantly higher in subjects with MetS than in those without MetS, and CeO was a predominant characteristics in subjects with MetS. Additionally, the prevalence of diabetes was significantly higher in subjects with MetS than in those without MetS. However, there was no significant difference in the prevalence of diabetes according to the presence of CeO among the MetS subjects. Accordingly, it may be that CeO is not an essential component for the diagnosis of MetS with respect to the identification of the risk for type 2 diabetes development in Asian populations. Considering that the fasting glucose level was significantly higher in MetS subjects without CeO than in those with CeO, and that fasting glucose was independently associated with increased baPWV in subjects with MetS, diabetes might have been less well controlled in MetS subjects without CeO than in those with CeO in this cohort study.

A number of previous studies have reported an inverse association between obesity and arterial stiffness in youth or middle age [39–41]. However, there is a paucity of data on this inverse association in an older population. Here, we identified an inverse association between CeO and arterial stiffness in relatively older subjects with MetS. Considering the results of a recent study that showed different component clusters of MetS revealed varying associations with arterial stiffness [42], our findings may provide substantial information on the association between individual MetS components and subclinical atherosclerosis in an older general population.

This study has some limitations. First, although the criteria of MetS might be dependent on ethnicity [43], the present study included only a Korean population. However, this might be a unique point for identifying the limitation of the IDF definition for predicting subclinical atherosclerosis in Asian population. Second, the impact of MetS on the progression of subclinical atherosclerosis might differ somewhat according to age group [44]. However, no sub-analysis according to age group was performed because the participants in this cohort study were relatively older. Third, several previous studies of Korean population reported that men had higher baPWV values compared with women [45, 46]. In the present study, the incidence of male gender was significantly higher in MetS subjects without CeO than in those with CeO, and this may be a clinical characteristic of atypical MetS subjects who do not have CeO. Although we identified an inverse association between CeO and baPWV in MetS subjects after adjusting for gender differences, further investigations are necessary to identify the impact of gender on this inverse association. Lastly, although the incidence of current medications for the treatment of hypertension, dyslipidemia, and diabetes was not significantly different between MetS subjects with and without CeO, we could not eliminate the possible effects of underlying medications on subclinical atherosclerosis because of the observational design of this study. Further large prospective studies are required to address these issues.

## Conclusion

An inverse association between CeO and arterial stiffness was observed in MetS subjects among relatively healthy Korean populations. This association persisted after adjusting for confounding CV risk factors. Moreover, the prevalence of diabetes was not significantly different in MetS subjects with and without CeO. In contrast to the IDF definition, the diagnosis of MetS may be important in Korean subjects without CeO but having multiple metabolic risk factors.

## Abbreviations

AMI: Acute myocardial infarction
BMI: Body mass index
baPWB: Brachial-ankle pulse wave velocity
CeO: Central obesity
CV: Cardiovascular
CVD: Cardiovascular disease
DBP: Diastolic blood pressure
HDL: High-density lipoprotein
IDF: International Diabetes Federation
LDL: Low-density lipoprotein
MetS: Metabolic syndrome
NCEP–ATP: National Cholesterol Education Program–Adult Treatment Panel
SBP: Systolic blood pressure
WHO: World Health Organization.
